# Healthy for Life Pilot Study: A Multicomponent School and Home Based Physical Activity Intervention for Disadvantaged Children

**DOI:** 10.3390/ijerph16162935

**Published:** 2019-08-15

**Authors:** Karma Pearce, James Dollman

**Affiliations:** 1 School of Pharmacy and Medical Sciences, Division of Health Sciences, Alliance for Research in Exercise, Nutrition and Activity (ARENA), University of South Australia, Adelaide 5001, SA, Australia; 2 School of Health Sciences, Division of Health Sciences, Alliance for Research in Exercise, Nutrition and Activity (ARENA), University of South Australia, Adelaide 5001, SA, Australia

**Keywords:** multicomponent home based physical activity program, MVPA, subjective measures PA, socially disadvantaged, enjoyment, self-management, self-efficacy, school support, social support, resilient

## Abstract

The study aimed to develop and evaluate a multicomponent school and home based physical activity (PA) intervention in children in grades 3–7 (aged 8–13 years) and determine the psychological variables that influence PA; 10 × 1 h school-based training sessions, a home-based activity program and 4 × 1 h lifestyle workshops for parents. PA was assessed at an intervention and nearby control school using accelerometers and self-report at 3-time points: baseline, post intervention and 10-week follow-up. Self-efficacy, self-management strategies, enjoyment, perceived barriers to PA, outcome-expectancy and social support were evaluated. The study showed 73% of the children with complete data sets at the intervention school (n = 27) did not increase device measured moderate to vigorous PA (MVPA) in the after-school period (3 p.m. to 6 p.m.) or over the whole day or during school break time immediately following the intervention or at follow-up, as compared to 70% of children with complete data sets at the control school (n = 35; *p* > 0.05 for all). Overall, 59% of boys attained more than double the recommended 120 min of MVPA each day compared to 42% of girls (*p* = 0.013). At the baseline, children’s self-reported PA in the intervention school positively correlated with: outcome expectancy (R = 0.240, *p* = 0.015), enjoyment (R = 0.339, *p* < 0.001), self-efficacy (R = 0.399, *p* < 0.001), self-management (R = 0.617, *p* < 0.001), social support at home (R = 0.406, *p* < 0.001), and social support at school (R = 0.407, *p* < 0.001). Similar relationships were observed after the intervention and at follow-up. Focus groups with the children, parents and interviews with teachers identified areas for improvement of the intervention. In conclusion, while the multifaceted approach to improve PA was ineffective over the time span of the study, important predictors of PA in this sample of disadvantaged children were identified.

## 1. Introduction

Insufficient physical activity (PA) is one of the ten leading risk factors for death worldwide [1]. This is not an issue confined to the adult population, with the estimated global number of overweight or obese children increasing from 32 million in 1990 to 42 million in 2013. Furthermore, this figure is projected to reach 70 million by 2025 [1]. The World Health Organization currently recommends that children engage in at least 60 min of moderate-to-vigorous intensity physical activity (MVPA) per day [1]. Active Healthy Kids Global Alliance (AHKGA) compared 49 countries from six continents to assess global trends in childhood PA in developed and developing nations and concluded that PA levels and sedentary behavior for children in Australia were some of the worst in the world, in contrast to levels in Zimbabwe and Japan where active transport is attributed to higher levels of PA and Slovenia where a high cultural emphasis is placed on sport [2]. The 2018 Active Health Kids Report Card showed on average 52% of Australian primary school aged child (5–13 years) attained at least 60 min of MVPA each day, with self-report data indicating a decrease to 17–40% of children meeting the guidelines each day [3]. Alarmingly, these figures have remained static over the 4-year period of monitoring. In contrast, only a third of similar aged children in nearby New Zealand failed to meet these guidelines [4].

Children who meet the minimum amount of PA every day have reduced risk of lifestyle diseases including overweight or obesity, Type II diabetes and metabolic syndrome, are aerobically fitter, have better musculoskeletal and bone health, and experience positive mental health benefits including a reduction in depression, stress, anxiety, and improvements in self-confidence and self-esteem [5]. Collectively, these benefits are critical for health, growth and development, particularly in preventing chronic disease and optimizing cardio metabolic health. Therefore, childhood should be viewed as an important ‘window in time’ for forming PA and sedentary behavioral habits that can track through to adulthood [6].

A recent review suggests an increasing prevalence of obesity in over half the studies among disadvantaged children and adolescents, compared to a third of studies among children and adolescents with a relatively high socioeconomic position [7], with these inequalities likely to contribute more broadly to socioeconomic inequalities in health [8]. The findings are also apparent in Australian children, with the most disadvantaged quintile attaining 67 min of MVPA each day, compared to those in the most advantaged quintile, who averaged 75 min each day [9]. However, it is generally accepted that improving participation in PA in socioeconomically disadvantaged groups is challenging, and in some cases, population-wide public health strategies which have failed to address the barriers of socioeconomic disadvantage have further increased inequalities [10]. Therefore, PA interventions that target socioeconomically disadvantaged children, while also addressing the barriers to socioeconomic disadvantage, are needed to improve population health outcomes over the longer term [11].

Enjoyable PA behaviors established early in life are most likely to be sustained over the longer term [6], and these behaviors are most likely to be influenced by families, schools, community based organizations and government agencies [12]. In most countries children spend almost half the year at school, and as much as 8–9 h contact a day, therefore the school sector has an enormous potential to influence, encourage and support the involvement of children in PA [13]. Earlier reviews on the effect of school-based PA interventions have shown mixed results [14], with reviewers highlighting issues with poor study design, lack of baseline data, and lack of control groups, PA measures of unknown reliability or validity and underpowered studies. Recent improvements in methodological quality, accompanied by device measured assessment of PA and appropriate statistical analyses, provide stronger evidence that school-based interventions can have a positive effect on PA [15]. A range of interventions have increased the amount of MVPA within PE lessons [16] and in recess and lunch periods [17], however some suggest that children may be compensating by being less active outside these windows [18]. The after-school period, commonly defined as 3 p.m. until 6 p.m., has been estimated to account for 21 to 48% of children’s MVPA, suggesting it should also be a vital window for investigation [19]. However, it is worth noting that interventions focusing just on increasing PA during the after-school time period have showed poor results, due to poor design, lack of power and implementation problems [20]. A review of the reviews focusing on after-school PA interventions in children and adolescents concluded there was modest support for the effectiveness of after-school programs on children’s PA levels, however, the overall evidence remains inconclusive and methodological differences were cited as a limitation in the review [21]. Additionally, Singletary et al. reviewed the effectiveness of school-based programs to increase PA, and concluded that current programs were insufficient to increase PA to achieve 60 min of MVPA/day (reviewed in [22]).

The Centers for Disease Control and Prevention suggest a multi-component comprehensive school PA program (CSPAP) involving quality physical education, PA during the school day as well as before and after-school, school staff (teacher) involvement in parallel to family and community involvement, offers one of the most successful approaches in improving PA levels in children [23,24]. Programs and policies to increase PA using multicomponent approaches through schools are currently being implemented in numerous countries, such as Ireland, Finland, France, Germany, and Switzerland [25]. A recent review of school-based programs aiming to increase PA amongst disadvantaged children found that multicomponent school-based health education programs which were comprised of culturally appropriate PA, a choice of activities which aimed to enhance motivation along with parent involvement, were all potential factors related to higher PA levels among students from disadvantaged background [26].

To date, very few researchers have used environmental based constructs or social cognitive theory to predict PA in socially disadvantaged children. Martin and colleagues used social cognitive variables in minority groups and found these variables were able to predict approximately 10% of the variance in PA [27,28,29]. Research in Arab American and African American children reported that perceived barriers to PA predicted PA [28,29]. Beets el al. (2007) report perceived barriers to PA are of importance to rural adolescent girls [30], while others report no associations at all [31]. Social support from both parents and peers were also important, with children from high income families receiving more support from siblings for PA and active play [32]. As children progress towards adolescence, the evidence suggests peers have a strong influence on the amount of PA attained [30]. Self-efficacy is arguably the most researched construct, possibly because it is a key construct in a number of health behavior change theories: social cognitive theory, protection motivation theory, trans theoretical theory and the theory of planned behavior [33]. Although work in minority groups is limited, researchers also report that, as a child’s self-efficacy increases so does their level of PA [27]. The most recent meta-analysis, examining 16 cross sectional studies and 7 longitudinal studies in adolescents of varying levels of socio-economic status revealed that 38% of the variance in PA could be explained by social cognitive variables; however, most of these studies have been conducted using self-report measures of PA [34]. Hence, there is a need to investigate the impact of these variables on device measured as well as self-reported PA, particularly among the social disadvantaged.

A multi-component comprehensive school and home based behavioral lifestyle program was developed for a socioeconomically disadvantaged school in consultation with all stakeholders (principal, teachers, parents and children) to target PA for the children and their families along with a series of lifestyle workshops for parents focusing on nutrition and PA. The study aimed to develop and evaluate a multicomponent school and home based physical activity (PA) intervention in children in grades 3–7 (aged 8–13 years). The primary aim was to evaluate whether ‘active play’ in children in grades three to seven in a socially disadvantaged school, particularly in the ‘after-school’ 3 p.m. to 6 p.m. period increased device measured and self-reported PA. Based on the school’s belief that the children within their community possessed lower levels of PA, we hypothesized that PA would increase. The secondary aims were twofold; to evaluate the effectiveness of a training program consisting of healthy lifestyle choices through nutrition and PA in the students’ parents or caregivers. We hypothesized that knowledge of healthy lifestyle choices and PA would increase in the parents and care givers. Finally, we aimed to explore and relationships between PA and the psychological variables that may influence PA in this cohort.

## 2. Materials and Methods 

### 2.1. Intervention Design

A nutrition and physical activity program for the children and their parents and caregivers was trialed in a socially disadvantaged school at the request of the school, in response to a perceived lack of knowledge about healthful nutrition and the need for adequate PA within their school community. The Stephanie Alexander Kitchen Garden program (SAKG), the leading nutrition primary school-based program in Australia provided financial support to create the kitchen garden and associated nutrition resources [35]. The SAKG, linked to the Australian weekly school curriculum, embraces a holistic approach to influencing children’s (nutritional) food choices by teaching them to grow, harvest, prepare and share fresh seasonal produce.

A multilevel school and home based behavioral lifestyle program, generally considered the most successful approach [23,24], was developed in consultation with all stakeholders (teachers, children, parents and the 2014 Principal at the intervention school) to target PA for the children and their families and a series of lifestyle workshops for parents focusing on nutrition and PA. This program was then reviewed by Department for Education and Child Development (DECD). The intervention was designed according to evidence that the most effective priorities for engaging children in PA are driven by the ‘desire to learn and improve skills, have fun, and develop friendships and affiliations’ [4] and it addressed the PA needs of children and their families. The intervention had 3 key components:

1. 10 × one-hour school-based training sessions consisting of 15–20 min of classroom training, linked to the National Health and Wellbeing curriculum [36] and 40–45 min of physical activities to support the training were delivered by a teacher and physiotherapist. The physical activities were, wherever possible, fruit and vegetable themed to complement the SAKG program [35]. The school-based sessions are outlined in more detail in Table 1.
2. a ‘passport to fun’ home-based activity booklet to encourage children’s daily participation in PA through ‘fun’ with their parents, guardians, siblings and friends over the 10-week intervention. The activities were designed to meet the needs of both small and large families and for parents and guardians who may have varying levels of physical ability and fitness. Activities consisted of a range of indoor and outdoor activities, and activities with and without equipment.
3. 4 × one-hour parent workshops, to support the SAKG program and promote awareness of a healthy diet and the importance of exercise, were delivered by a trained nutritionist and educator. All sessions were based on the Australian Guide to Healthy Eating (AGHE) and the Australian PA guidelines for all Australians (12). Instructions were given on: how to read a food label to identify hidden fats, sugar and salt; food groups and serving sizes; and nutrition and PA for disease prevention. Parents from the intervention school were invited through the school newsletter, Parents and Friends Association and individual flyers to attend 4 × 1 h workshops on the benefits of a healthy diet and PA. Attending parents were provided with a demonstration of how to cook a healthy main course and dessert for their household. They were provided with enough food to prepare the meal for all those eating within the family home, with instruction and food free of charge. They were invited to provide feedback on the workshops and complete the Australian Nutrition Knowledge Questionnaire (GNKQ) before and after training (13) and a short questionnaire on PA.

### 2.2. Participants

All students aged 8 to 13 years in two socially disadvantaged primary schools in the northern suburbs of Adelaide, Australia were invited to participate. The two schools were demographically comparable due to similar location, enrollment numbers, physical environment and facilities. The schools were located 1.6 km apart and were both ranked by the Department of Education and Child Development (DECD) as Category 2 schools on the Index of Educational Disadvantage, where Category 1 is the most disadvantaged and Category 7 the least disadvantaged. Both schools had kitchen gardens and offered the SAKG program [35] as part of the curriculum (DECD 2014; (10)). Parents of consenting children from both schools provided socio-demographic data. Parents from the intervention school were invited through the school newsletter, Parents and Friends Association and individual flyers to attend 4 × 1 h workshops on the benefits of a healthy diet and PA. Staff at the intervention school was interviewed about the program at the end of the intervention. Data collection occurred between March and November 2015, with the intervention occurring over autumn (fall) and winter and the follow-up period over winter and spring. Ethical approval was obtained from the University Human Research Ethics Committee (no: 0000033710) and DECD (CS/14/511-39). Written consent was obtained from the principal in each school and from parents through a letter explaining the study goals, procedures and methods.

### 2.3. Study Design

**Figure 1:**
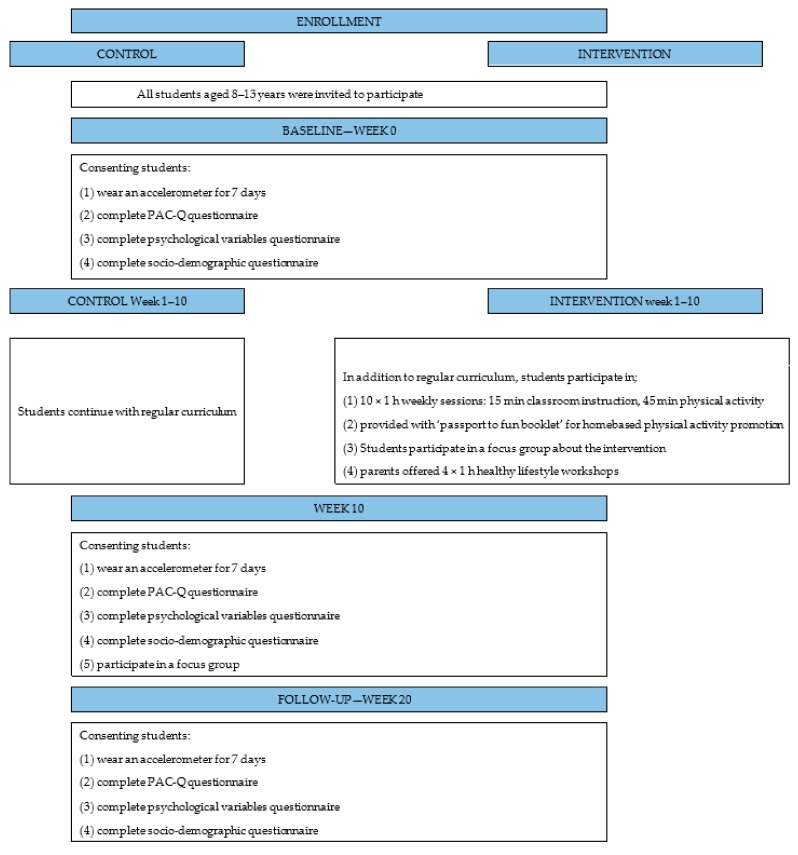
Research design and measures.

All participants providing consent from both schools were invited to provide measures of psychological variables related to PA and device measured (accelerometer) and self-reported PA. Parents of consenting children from both schools provided socio-demographic data. All students within grades 3–7 (8–13 years) enrolled in the intervention school received the school-based activities (n = 98 at baseline), however only children providing consent participated in the home-based ‘passport to fun’ activities with their families (n = 63). The study design is outlined in Figure 1.

### 2.4. Measures

#### 2.4.1. Psychosocial Variables

Psychological variables were evaluated to determine their impact on PA. Self-efficacy was measured using a validated eight-item questionnaire (e.g., I can be physically active during my free time on most days) developed for use with children aged 10 to 15 years [37,38,39].

Self-management strategies were measured using an abridged version of the validated survey initially developed from self-management theory for use with graduate students. The scale consisted of 6 items (e.g., I think about the benefits I will get from being physically active) [39,40].

Enjoyment of PA was assessed by a validated abridged version of the Physical Activity Enjoyment Scale and used 6 negatively worded items (e.g., I feel bored) ranging from disagree a lot to agree a lot, with a low score representing a low level of enjoyment of PA [37].

Perceived barriers to PA were derived from a validated adaptation of a 9-item measure developed for the Trial of Activity for Adolescent Girls (TAAG) pilot study [39]. Items rated the level of perceived confidence of the participant to perform regular PA in the face of a particular barrier (e.g., “If it is bad weather” or “I’m chosen last for teams”) with low scores represented large perceived barriers.

Similarly, the outcome-expectancy value of PA developed by Dishman et al. (2010) was measured as the average of 9 belief statements (e.g., it would help me spend more time with my friend) and their corresponding value statements (e.g., Spending more time with my friends is…).

Social Support from both teachers and family and friends was measured using two correlated scales from the Amherst Health and Activity Study [41].

#### 2.4.2. Physical Activity

PA was assessed at an intervention and nearby control school using accelerometers and self-report at 3-time points: baseline, post intervention and 10-week follow-up.

##### Self-Reported Physical Activity

Participants completed the validated Physical Activity Questionnaire for Children (PAQ-C) on three occasions. This questionnaire is a validated seven-day self-report PA measure, consisting of nine items which are used to determine summary activity scores over the last week [42]. Items assessed PA performed at school break time, immediately after-school, and at home.

#### 2.4.3. Device-Measured Physical Activity

In order to directly measure PA, children wore an ActiGraph GT3X+ accelerometer (ActiGraph, Ft. Walton Beach, FL, USA) during waking hours, every day, for a week. Children were instructed on how to wear the monitor, were fitted with the accelerometers on their right hip and provided with instructions on how to wear and also care for the accelerometer. The accelerometer was pre-programmed to collected data at 5 s intervals. At the completion of the 7 days, the accelerometers were collected and data downloaded. The minimal amount of accelerometer data that was considered acceptable was 4 days with at least 8 h of waking-hours of wear time per day, including at least one weekend day. Using cut points of sedentary activity <100 counts.minute^−1^(cpm); light activity 100 to ≤2296 cpm; moderate activity >2296 to ≤4012 cpm; vigorous activity >4012 cpm) [43], the average minutes spent in moderate-to-vigorous activity over the entire day were calculated. The average daily time spent in light, moderate and vigorous activities during the weekday “after-school” period (3 p.m. to 6 p.m.) [19] and during recess (30 min at the intervention school, 20 min at the control school) and lunchtime (20 min at the intervention school, 30 min at the control school) were determined for days that met the minimum inclusion criteria of counts recorded at break time. Recess and lunch time PA data were summed to provide the total time children engaged in MVPA during daily school break time (total 50 min) and averaged per valid day. The percentage of time children engaged in this activity was determined by dividing the time spent in each of these intensities by total available time during breaks [44]. Children returning accelerometers were provided with toys (approximately $AU5).

#### 2.4.4. Assessment of Parent Information Sessions.

##### Nutrition Knowledge

Nutrition knowledge was assessed using a self-administered validated General Nutrition Knowledge Questionnaire (GNKQ-Aust) [45]. The GNKQ-Aust consisted of 113 questions that assessed understanding of general nutrition-related terminology; awareness of current dietary recommendations (13 items), knowledge of food sources related to nutrients (70 items), the use of dietary information to make dietary choices (10 items), and an awareness of the relationship between diet and disease (20 items). Each correct answer to a question was worth 1 point. The questions were compatible with the Dietary Guidelines for All Australians (adult version). Each participant’s responses were manually coded numerically and converted to a corrected score, as defined by Parmenter and Wardle [46]. All questions were equally weighted with a maximum of 13 points awarded for knowledge of dietary recommendations, 70 for sources of nutrients, 10 for choosing everyday foods, and 20 for the diet-disease relationships, for a possible total score of 113. A higher score reflected a higher level of knowledge.

##### Assessment of PA Knowledge

Parents were asked to record the number of minutes of exercise per day recommended for primary school aged children, adolescents and adults. In an open ended question, they were asked to comment on the health benefits of meeting the requirements at any age.

#### 2.4.5. Program Feedback

At the completion of the program, the parents and children participated in a focus group and the students, deputy principal and teachers were interviewed to determine areas for program improvement.

##### Focus Groups with the Children

As a class group, students responded to the following questions: what aspects of the program did you like best?, did you make up any active play activities yourself (yourselves)?, what aspects of the program did you like least?, did anything prevent you from enjoying the class based activities or homework activities? and finally, was there any part of the program you would like us to change?

##### Focus Groups with the Parent

Feedback was sought from the attending parents on the features of the program(s) they enjoyed most, the aspects they enjoyed least, the barriers or aspects that may have prevented them from engaging or enjoying the educational program or homework-based activities and any aspects that felt should be removed or changed.

##### Interviews with the Teachers

At the completion of the program, teachers were asked which year level they taught and the number of children in their classroom. They were also asked to comment on the following characteristics about their class at the completion of the healthy for life hourly sessions delivered during class time: effect on awareness of the importance of PA and healthy lifestyles; appropriateness for the year level; effect on the children’s antisocial behavior, the children’s’ attention/concentration, the children’s cognitive function; effect on the children’s confidence in participating in PA; and the children’s social connectedness. They were then invited to suggest how engagement with the homework ‘passport to fun’ activities could be improved and compliance with accelerometer wear time could be improved.

### 2.5. Statistical Analysis

Statistical analyses were conducted using IBM SPSS software, version 20 (SPSS Inc., Chicago, IL, USA). All variables were reported as mean ± standard deviation. Correlations were assessed using the Pearson’s method. The impact of the intervention on measures of PA was analyzed by repeated measures analysis of variance (ANOVA) with ‘school’ as a between-subject factor and ‘time’ (baseline, post-intervention and follow-up’) as a within-subject factor. Adjustments were made for sex, class, and parents’ marital and work status as covariates. Bonferroni-corrected independent samples *t*-tests were computed to compare PA in intervention and control schools at the different sampling points if the ANOVA revealed a significant treatment × time interaction. Statistical significance was set at *p* < 0.05.

## 3. Results

### 3.1. Participants

**Figure A1:**
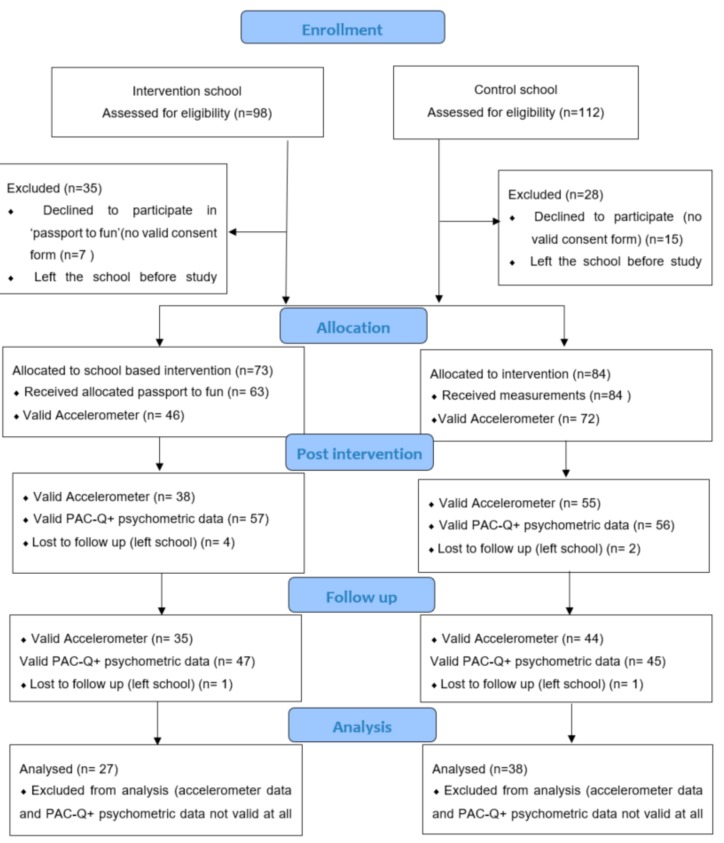
Consort Diagram.

Consent forms were received from 63 students enrolled at the intervention school and 84 students in the control school with the proportion of male and female students in classes the same across both schools (*p* > 0.05). However, only 27 students at the intervention school and 38 students at the control school completed all aspects of the study (including valid accelerometer wear times). Lost accelerometers (2 at the intervention school and 3 at the control school) accounted for a small proportion of missing data with the majority due to low wear time. Additionally, between planning the study and study implementation, 19% and 12% of students left the intervention and control school respectively, with a further 5 students leaving the intervention school and 3 leaving the control school during the study. A consort flow diagram is available as Appendix A Figure A1. Overall, the children were equally distributed across targeted year levels, with the intervention school having a higher percentage of Aboriginal children (*p* = 0.021), unemployed parents or carers (*p* = 0.032) and a trend towards more single parent families (*p* = 0.082). Only 2 of the parent or carer respondents from either school had completed post high school study. The characteristics of the children are shown in Table 2.

In relation to the parent sessions, seven parents or carers attended at least one workshop with only 3 families (4 adults) represented at all workshops. The average age of the 4 women, all of European descent, was 40 ± 19 years. One woman was widowed, one married and two were single parents. Only one woman worked 4 h per week in paid work. Three women had attained a high school education and the other completed a primary school education (a grandmother attended with a mother).

### 3.2. Physical Activity Levels Achieved through Active Play

Device measured daily PA (MVPA) showed children at both schools undertook similar levels of PA with no effects of time (baseline, post–intervention or follow-up; *p* > 0.05 for all) suggesting that the intervention (including the 1 h school based intervention and after-school activities) was not effective in improving time spent in MVPA (mins) across the day.

The ‘passport to fun’ intervention aimed to target and increase PA levels in the after-school period (3 p.m. to 6 p.m.), yet no increases were observed post intervention and at the 10-week follow-up period (*p* > 0.05) for both. No additional increases were observed in break times (>50% MVPA over 50 min) during the school day at either school or at any time point (*p* > 0.05 for all). Table 3. Taken together, these results suggest that the class-based activities and ‘passport to fun’ homebased activities were not effective in increasing device measured MVPA in the after-school period, or across the day or a school break times. One explanation for this may be the initially high levels of initial PA of the children; on average 72% of the 65 children at both schools that completed all aspects of the study achieved more than the recommended 60 min of MVPA each day (73% at the intervention school and 70% at the control school) and 49% of children achieved more than 120 min of MVPA each day at baseline (*p* > 0.05).

As there were no significant differences in the level of MVPA over the day, at break time, or in the after-school period (3 p.m.–6 p.m.) between schools or within schools post intervention or at the 10-week follow-up, the data were analyzed as a single cohort in subsequent analyses to increase statistical power to examine the effects of sex and class level. Overall, there were no differences in the proportion of boys and girls achieving the recommended 60 min of MVPA each day (*p* > 0.05), but more boys attained more than 120 min of MVPA each day compared to girls (59% vs. 42%; *p* = 0.013). The class level had no impact on the level PA achieved (*p* > 0.05). Overall, only 59% of the children achieved more than 50% of recess time in MVPA. There were no effects over time for children spending more than of the 50% of break time in MVPA or between schools (*p* > 0.05 for all). Similar effects were observed with the amount of time spent in MVPA after-school (3 p.m.–6 p.m.; *p* > 0.05 for all).

Self-reported levels of PA are outlined in Table 4. The intervention school children reported slightly higher levels of self-reported PA compared to the control school between baseline and post intervention (3.4 ± 0.8 vs. 3.2 ± 0.8; *p* = 0.042) and after the 10-week follow-up (3.7 + 0.6 vs. 3.5 ± 0.6; *p* = 0.009), although the effect sizes were small (0.043–0.089).

### 3.3. Relationships with Device Measured PA and Self-Reported PA

In the intervention school at baseline, there was a significant relationship of moderate effect size between the device and self-reported measures of PA (R = 0.450, *p* = 0.006) which was maintained after adjustment for gender (R = 0.341, *p* = 0.008), suggesting acceptable concurrent validity between the measures. Similar levels of significance were achieved after the intervention and at follow-up and at the control school at all time points.

### 3.4. Psychological Variables which Influence PA

Comparing psychological measures at baseline, post intervention and at the end of the follow-up period; there were no significant differences in any of the psychological measures (self-management strategies, perceived barriers to PA, outcome-expectancy value of PA, enjoyment of PA, self-efficacy and social support from both family and friends and the school) at baseline, after the intervention or at follow-up within the intervention school (*p* > 0.05 for all) or the control school (*p* > 0.05 for all).

Overall, there were between school effects with significant differences between social support from both family and friends (*p* < 0.001) and the school (*p* < 0.001) and enjoyment (*p* = 0.007) between the intervention school and the control school, although these differences were small and represent a variability of 0.3 to 0.5 units, Table 4.

#### 3.4.1. Psychological Variables which Influence Device Measured PA

At baseline, for children in the intervention school, device measured PA positively correlated with: enjoyment (R = 0.215, *p* = 0.043), self-efficacy (R = 0.330, *p* = 0.025), self-management (R = 0.508, *p* < 0.001), social support at home (R = 0.276, *p* = 0.033) and social support at school (R = 0.376, *p* = 0.026). Effect sizes improved after adjustment for sex and school class, ranging between 0.272 and 0.394. Similar relationships were observed after the intervention and at follow-up. Similar trends were observed at the control school; at baseline, children’s self-reported PA positively correlated with; enjoyment (R = 0.390, *p* = 0.002), self-efficacy (R = 0.448, *p* < 0.001), self-management (R = 0.586, *p* < 0.001), social support at home (R = 0.550, *p* < 0.001) and social support at school (R = 0.551, *p* < 0.001). Effect sizes improved after adjustment for gender and school class ranging between 0.394 and 0.593. Similar relationships were observed after the intervention and at follow-up.

#### 3.4.2. Psychological Variables which Influence Self-Report PA

At the baseline, for children in the intervention school, self-reported PA positively correlated with: outcome expectancy (R = 0.240, *p* = 0.015), enjoyment (R = 0.339, *p* < 0.001), self-efficacy (R = 0.399, *p* < 0.001), self-management (R = 0.617, *p* < 0.001), social support at home (R = 0.406, *p* < 0.001) and social support at school (R = 0.407, *p* < 0.001). Effect sizes improved after adjustment for sex and school class, ranging between 0.362 and 0.512. Similar relationships were observed after the intervention and at follow-up. Similar trends were observed at the control school; at baseline, children’s self-reported PA positively correlated with; outcome expectancy (R = 0.295 *p* = 0.021), enjoyment (R = 0.390, *p* = 0.002), self-efficacy (R = 0.448, *p* < 0.001), self-management (R = 0.499, *p* < 0.001), social support at home (R = 0.550, *p* < 0.001) and social support at school (R = 0.551, *p* < 0.001). Effect sizes improved after adjustment for gender and school class ranging between 0.394 and 0.543. Similar relationships were observed after the intervention and at follow-up.

### 3.5. Feedback from all Stakeholders; The Children, Parents and Teachers

Feedback from the children was obtained for both the class-based and ‘Passport to fun’ home-based activities through class-based focus groups; within the school, older children preferred ball-based games and the opportunity to create their own games, whereas the younger children just liked ‘playing’. Interestingly, older children preferred more ‘outside time’, yet were more critical of the activities per se. In relation to the ‘Passport to fun’, older children again preferred ball-based games, while the younger children appreciated the variety of activities provided in the booklet. Over a third of students valued the opportunity to use the ‘passport to fun’ to gain ideas of games to play with their family or friends; ‘if there’s nothing on TV or nothing to do you can do something from the book’, or ‘it gives you ideas of things you can play with your friends’. However, just under a third of predominantly older children stated they had never used the booklet citing, PA is ‘boring’, ‘not fun’ or in the case of 3 children, ‘I can’t read’. These responders were then asked to provide feedback on what they would like to see changed; no constructive feedback was supplied. Students who were more positive about the ‘passport to fun’ wanted more activities they could do by themselves, suggesting a number of children completed the passport activities without parental/carer support. Barriers to participation included bathing/showering (14%), going to bed (11%), participating in sport (17%), playing with or feeding animals (19%), bad weather (14%) or forgetfulness (31%).

The school staff was approached to provide feedback on the school and home-based activities; two of the four teachers and the deputy principal provided feedback. The teachers believed the program promoted an increased awareness of the importance of PA and healthy lifestyles and were generally appropriate to the year levels. They suggested the best feature of the school-based activities were the PA based games, as they were seen as ‘fun’ and promoted teamwork and social interactions. Both teachers reported ‘some’ positive changes in the children’s attention, concentration, confidence to participate in PA, and antisocial behavior. Teachers believed the homework component would be beneficial in other schools, but generally not their own school and suggested possible reasons for the lack of engagement with this element of the program were ‘lazy’ students, unwilling parents or parents with low levels of literacy. Teachers consistently set homework, with reported completion rates of 30–80%, depending on the task and the teacher. The generally low completion rates suggest family support was low and may in part explain the lack of engagement with the ‘passport to fun’.

The teachers were invited to comment on the compliance with accelerometer wear-time; the teachers believed the information provided just prior to wear-time and in one case, daily prompting from the teacher, encouraged students to wear the accelerometers correctly. However, less than 7% of the children wore them for the current minimum standard of more than 10 h each day and 4 week days and one weekend day [48]. Teachers suggested possible reasons for this were the lack of support from parents, and forgetfulness of the students, suggesting incentives at all time points may increase compliance. One teacher suggested target goals based on the number of steps per day accompanied by real time feedback may increase the engagement of older children. Another suggested sending a note home to parents seeking their commitment to monitoring and supporting the program (although this was outlined in the information sheet). Another acknowledged the parent sessions further encouraged the engagement of parents with their children in the ‘passport to fun’, but recognized that this was limited by the 3 families represented at these sessions.

Feedback was also sought on the parent sessions. Attending parents were very supportive of the workshops and indicated they would have attended irrespective of the ‘free food’ to prepare healthy recipes for their household, as they were very interested in improving their lifestyles. These parents also reported actively promoting these workshops through the Parents and Friends Association and the school yard. All parents acknowledged the reminders in the school newsletter. Specific feedback indicated that the sessions were very informative and were delivered in a relaxed atmosphere that promoted and supported questions from participants. They particularly liked learning to read ‘food labels’ and learning to ‘myth bust’ foods that are marketed as healthy. The participants reported 15% increase in the overall knowledge of nutrition (assessed via the GNKQ (13)) and the importance of PA assessed via additional questions on the required amount of daily PA with all adults correctly reporting PA levels for adults and children and were aware of the health benefits associated with adequate PA.

## 4. Discussion

Schools are considered an ideal environment to implement strategies and interventions to promote PA and are considered among the most influential avenues for supporting and encouraging children to participate in the recommended amount of time engaged in PA [24]. Once approached by the primary school, a multifaceted program to promote family-based PA based on best practice was created after consultation with all stakeholders; including teachers, parents/guardians and children, to provide supportive programs and environments in a ‘whole school community approach’, with the money it had available [24]. In contrast to many previous studies which have evaluated school-based interventions in terms of PA during the school day [43,49], this study sought to also understand the influence of psychological variables on PA.

The key findings from this study initiated by the intervention school showed that the multifaceted approach using classroom-based activities, a series of parent workshops and a ‘passport to fun’ home based activity booklet to promote PA outside this socially disadvantaged school was not successful in improving PA either post intervention or at the 10-week follow-up period. There are several possible explanations for this. First, while a number of researchers report that children from low socio-economic backgrounds are significantly less active compared to those of high socio-economic backgrounds (reviewed in [50]), others recognize that some children in that group are physically active [51]. Two large Australian studies exemplify this. A study of 460 primary school aged children from disadvantaged socio-economic back grounds spent an average of 54.8  ±  19.7 min per day in MVPA [50], while the other in 373 boys and girls reported levels of 195 ± 67.8 and 156.4 ± 62.9 min per day of MVPA respectively in which 84% of children met current PA recommendations [51]. The children in the second study were described as ‘resilient’, as they appeared to resist the increased odds of sedentary lifestyles typically associated with socioeconomic disadvantage [51]. In our study, the children averaged a total of 138.8 ± 45.5 min of MVPA over all time points with 72% of children achieving the minimum level of daily PA and 49% of children achieving more than double the minimum recommended MVPA level. Combined, this suggests the initial levels of MVPA were high leaving minimum room for improvement and the children could be seen as ‘resilient’ in the context of socioeconomic disadvantage. Second, while socially disadvantaged communities are often challenging environments to work in, Dai et al. (2019) cite one of the factors likely to influence the success of PA programs with socially disadvantaged children is parental involvement [26]. Lack of participation in the parent workshops, despite being heavily publicized through the school newsletters, individual flyers sent to every child’s home and the Parents and Friends Association, attendance may have been influenced by the announced closure of a manufacturing (car) plant, a major employer of many parents in the school community, with the resulting unemployment impacting on the level of engagement of the parents/guardians. The closure of the manufacturing plant also resulted in a large number of students leaving the school over the planning and implementation phases of the study which resulted in the study being under-powered and unable to detect meaningful change in PA.

School teachers and staff also form a key role in supporting and promoting multi-component comprehensive school PA program (CSPAP) [23,24]. A change in principal and key staff, including the kitchen garden coordinator also resulted in a cultural shift in the uptake of the program at the intervention school. Third, 77% of the students walked to and from school; this was 52% more than children in many other Australian schools (27). This increased PA could possibly have reduced motivation to engage in additional PA in the after-school hours period or over the day. Fourth, the percentage of children completing all aspects of the study (43%) was lower than expected compared to other Australian studies in similar populations (48–51%) with more stringent accelerometer inclusion criteria; a minimum of 4 valid wear days and one weekend day with 10 h of ‘wear time’ [50,52], which may have confounded the results. Finally, the parents within the intervention school community specifically requested a series of workshops on healthy food and PA with a focus on how to read a food label, identifying hidden fats, sugar and salt, food groups and serving sizes, and nutrition and PA for disease prevention. It was disappointing to only have 3 families complete all workshops, despite the fact that the sessions were advertised extensively and free food was provided to make an evening main course and dessert for all those eating within the family home that evening. Additionally, parents were asked to sign the ‘passport to fun’ booklets after their child completed any activities. Less than one in every 5 children returned booklets with signatures for 50% of the days over the intervention period, again highlighting the lack of commitment on behalf of the parents.

We believe that this is the first study to employ an extensive number of psychological tools (perceived barriers to PA, self-efficacy, self-management, enjoyment, outcome-expectancy and social support (from teachers and family/friends)) to examine the relationships between these variables and both device and subjective measures of PA in boys and girls in a socially disadvantaged ‘resilient’ primary school setting. A strong association was observed between both device measures of daily PA (MVPA) and self-reported PA and self-management in both the intervention school and control school at baseline, post intervention and follow-up. Furthermore, strong or moderate association were observed with both device measures of daily PA (MVPA) and self-reported PA with enjoyment, self-efficacy, home and school support at both schools, and at all time points suggesting these psychological measures are important to ‘resilient’ children from socially disadvantaged backgrounds. Self-efficacy theory is based on the theory that efficacy beliefs have both a direct and indirect effect on PA by influencing self-management and perceptions about the environments that either support or hinder PA. Similarly, perceptions of social support have been shown to be inversely related to self-efficacy and may influence PA through self-management [53]. Additionally, enjoyment has been linked to participation in school-based PA [41], so it is not surprising that these variables have been linked to PA in this study. However, neither device nor subjective measures of PA were predicted by perceived barriers to PA. This is in contrast to others who report social support and self-efficacy are mediated by perceived barriers to PA [54,55]. This may suggest that ‘resilient’ children might be readily able to overcome a number of barriers to PA which effect more advantaged children. However, given the small number of children in his study we suggest this finding warrants further investigation. It may also be important that future intervention studies in this cohort ensure schools provide adequate support for these children and employ strategies to also engage their parents.

The strengths of study included the extensive consultation process to design the intervention resources with all stakeholders, a review by DECD and the use of both device and subjective measures of PA. The study also employed the use of a control school which was of similar location, size, physical environment and facilities. However, the study was powered to recruit 224 participants into a parallel study (based on differences between baseline, post intervention and follow-up of 11 ± 33 MVPA minutes per day, with 80% power and alpha levels of *p* < 0.05 [15]. Between study design and study commencement, the number of students in years 3 to 7 at both schools dropped from a total of 241 to 68 at the intervention school and 87 at the control school, so it is possible that the study may have been underpowered to measure any change in MVPA. As the school had particularly sought a ‘home-based’ program to promote PA outside of school hours, it was particularly surprising to have current teachers state that, with the exception of reading, homework was not always expected or enforced, with accepted completion rates in some classrooms as low as 30%. With the exception of the year 7 students, the children generally enjoyed and actively participated in the school-based activities. However, only 18% of the children returned ‘passport to fun’ booklets which were signed by parents for more than 50% of the days during the intervention, further highlighting the lack of commitment of parents and guardians to the program.

Finally, we acknowledge the small number of teacher and parent responses to the focus groups is a limitation, however we believe their comments are of value and the low participation in the focus groups is reflective of how extremely challenging it is to engage the wider school community in health promotion initiatives such as the one represented in this study.

## 5. Conclusions

The current study found a multicomponent school and home based physical activity (PA) intervention in children in grades 3–7 (aged 8–13 years) consisting of school-based training sessions, a home-based activity program and lifestyle workshops for parents was not effective in increasing either self-reported or device measured PA in the children when compared to children enrolled at a nearby control school post intervention or at a 10-week follow-up. However, the children were found to have higher than expected baseline PA suggesting they were ‘resilient’ children. Importantly, in this socially disadvantaged ‘resilient’ group of children, outcome expectancy, enjoyment, self-efficacy, self-management, social support at home, and social support at school were found to be important predictors of PA and should be considered when planning similar interventions in this cohort.

The design of this multilevel program is firmly grounded in current theories, and in the right environment (children with low levels of PA) warrants further investigation, over a time period such as 3 to 5 years, to enable a cultural shift within families and the school environment. Based on teacher and student feedback, we acknowledge a need to modify the class-based activities for the year 7 students to increase student engagement and suggest future implementation of a similar program include ‘observers’ to pin point specific areas of weakness in the program and determine whether the development of motor skills may further encourage episodes of activity. Future studies will need to overcome barriers of parental, teacher and student engagement (possibly through incentives and more proactive promotion through assemblies and the local community) but will still be vulnerable to key staff changes. This may be overcome by the appointment of a health facilitator. Future studies should also concentrate on strategies that increase self-efficacy, self-management, and enjoyment and should funding permit, part of a broader investigation with nutrition and sleep.

## Figures and Tables

**Table 1:** School based intervention.

Week	Classroom Instruction		Outdoor Instruction
	Session Outcomes/Curriculum Link	Elaborations	
1	Identify and practice strategies to promote health, safety and wellbeing (ACPPS036)	What is physical activity? How much physical activity do you need in a day?	Team based games: rob the fruit bowl, run the gauntlet, rotten apples
2	Identify how to set a goal. Strategies for goal setting	Define goal setting and use SMART (s-specific, m-measurable, a-achievable, r-realistic, t-timely) goal format. Set goals for ‘passport to fun’.	Vegetable Olympics: carrot relay, vegetable tunnel ball, egg and spoon relay
3	Barriers and enablers to physical activity at home	Identify barriers and brainstorm enablers	Team building; build a scarecrow.
4	Barriers and enablers to physical activity at home	Identify barriers and brainstorm enablers	Gym stations: rope, mat, ball and cone activities
5	Examine the benefits of physical activity and physical fitness to health and wellbeing, (ACPMP046)	Benefits of physical activity and physical fitness to health and wellbeing to include the influence on sleep, concentration and fitness	Dance routines
6	Identify and practice strategies to promote health, safety and wellbeing (ACPPSO36)	Introduction to pedometers	Pedometer—‘treasure’ map around the edible kitchen garden located around the perimeter of the school.
7	Cultural considerations to physical activity, (ACPPS042)	Discussion on cultural diversity within the class and link to diversity in traditional sports around the world and in Australia.	Active vegetable and fruit memory games
8	The importance of Friendships and engaging friends in physical activity, (ACPMP048)	Discussion on the importance of friendships and enablers to building friendships	Capture the carrot tag game
9	Liquids while exercising	Discussion on water vs. sports drinks, energy drinks, cordial, fruit juice	Fruit salad soccer game
10	Reassess goals	Refer to ‘passport to fun’ and level of participation	Healthy ‘food plate’ relay

Table 1: The school-based intervention consisted of 15–20 min of classroom-based instruction (linked to the national curriculum where possible) and 40–45 min of outdoor PA based games.

**Table 2:** Demographic data.

Participants	Intervention School n = 63		Control School n = 84		All n = 147	*p* Values
	% Cohort	% Responders	% Cohort	% Responders	% Responders	
Female (%)	64	64	55	55	59	0.294
Class year (%)						0.265
combined 3/4	27	27	23	23	25	
combined 4/5	27	27	23	23	25	
straight 6 + combined 5/6	26	26	26	26	27	
straight 7	19	19	29	29	23	
Ethnicity (%)						0.755
Aboriginal	13	19	3	11	7	
European	14	21	6	26	10	
African	10	14	1	0	4	
Asian	0	0	4	16	2	
Other	31	47	11	47	20	
Highest parental education (%)						0.755
High school (partial or completed)	54	63	23	71	38	
Some post-secondary	29	33	8	23	17	
Bachelor degree	2	2	1	3	1	
Postgraduate	2	2	1	3	1	
Parent marital status (%)						0.082
married/partner	41	48	25	68	53	
single parent/guardian	44	52	12	32	45	
Parents working outside the home (%)						0.032
none	59	68	18	48	35	
<15 h/wk	13	15	2	7	7	
part time (15–35 h/wk	5	6	11	29	8	
full time (36+ h/wk)	9	11	6	16	8	
Household size (no. of people living in the house) mean (SD)	5.3 (2.6)		4.9 (1.5)		5.2 (2.9)	0.469

Data provided at baseline, due to incomplete reporting some data for % cohort do not add up to 100%.

**Table 3:** Device measured Physical Activity.

Timeframe MVPA (min/day)	Data at Each Time Point			Complete Data for 3 Time Points		
	Baseline	Post Intervention	Follow-Up	Baseline	Post Intervention	Follow-Up
Intervention School (n)	46	38	35	27	27	27
Total day	147 ± 44	138 ± 44	145 ± 42	146 ± 41	142 ± 8	143 ± 40
School break time	43 ± 6	41 ± 6	44 ± 5	43 ± 2	43 ± 2	42 ± 3
After-school	60 ± 6	58 ± 7	59 ± 6	59 ± 6	56 ± 7	59 ± 6
Control school (n)	72	55	44	38	38	38
Total day	131 ± 26	132 ± 33	131 ± 29	138 ± 36	132 ± 42	144 ± 40
School break time	42 ± 2	41 ± 4	43 ± 3	44 ± 3	43 ± 2	41 ± 3
After-school	56 ± 5	50 ± 6	51 ± 4	53 ± 5	49 ± 7	48 ± 6

Note: Minimum wear time was 8 h per day for a minimum of 4 days (19). MVPA; moderate-to-vigorous-intensity physical activity (≥4012 cpm) [43]. There were no statistical differences between any of the measures of PA at baseline, post intervention and follow-up within either school across the total day, during break time (recess and lunch total of 50 min (activity levels >50% break time) or during the after-school period (3 p.m. to 6 p.m.) or between the control and intervention school. MVPA (min/day), *p* > 0.05 for all.

**Table 4:** Subjective measures of Physical Activity and Psychological Variables.

Intervention School	Data at Each Time Point			Complete Data for 3 Time Points		
	Baseline	Post Intervention	Follow-Up	Baseline	Post Intervention	Follow-Up
n	63	57	47	27	27	27
PAC-Q	3.4 ± 0.8	3.4 ± 0.9	3.7 + 0.6	3.4 ± 0.8	3.6 + 0.6	3.7 ± 0.6
self-management	2.9 ± 0.7	2.8 ± 0.9	3.5 ± 0.6	3.2 + 0.8	3.0 + 0.9	3.5 + 0.6
barriers	2.0 ± 0.7	2.0 ± 0.7	2.8 ± 0.8	2.2 + 0.8	1.9 ± 0.5	2.8 ± 0.7
outcome expectancy	3.7 + 1.1	3.6 ± 1.1	2.5 ± 1.1	3.5 ± 1.2	3.9 ± 1.0	2.6 ± 1.3
enjoyment	3.2 ± 0.8	3.0 ± 0.7	3.5 ± 0.9	3.4 ± 0.9	3.1 ± 0.6	3.5 ± 1.0
self-efficacy	3.5 ± 0.9	3.5 ± 1.0	3.3 + 0.9	3.6 ± 0.9	3.7 ± 0.8	3.3 ± 0.9
social support home	3.0 ± 1.2	3.3 ± 1.0	3.6 + 1.0	3.1 ± 1.1	3.3 ± 1.1	3.5 ± 1.0
school support	3.0 ± 1.2	3.2 ± 1.1	3.3 ± 1.1	3.2 ± 1.1	3.3 + 1.1	3.3 + 1.1
Control school						
n	72	56	45	38	38	38
PAC-Q	^a^ 3.2 ± 0.8	3.6 ± 0.6	^b^ 3.5 ± 0.6	^a^ 3.1 ± 0.7	^a^ 3.3 ± 0.6	^c^ 3.3 ± 0.9
self-management	^a^ 3.2 ± 0.8	3.0 ± 0.9	^b^ 3.5 ± 0.6	^c^ 2.4 ± 0.8	^a^ 2.4 ± 0.8	^c^ 3.1 ± 0.7
barriers	2.2 ± 0.8	1.9 ± 0.5	^a^ 2.8 ± 0.7	1.8 ± 0.6	1.8 ± 0.7	1.7 ± 0.7
outcome expectancy	3.5 ± 1.2	3.9 ± 1.0	2.6 ± 1.3	3.3 ± 1.0	3.3 ± 0.9	^a^2.8 ± 1.6
enjoyment	3.4 ± 0.9	3.1 ± 0.6	3.5 + 1.0	2.9 ± 0.7	2.8 ± 0.6	3.5 + 0.9
self-efficacy	3.6 ± 0.9	^a^ 3.7 ± 0.8	^a^ 3.4 ± 0.9	3.3 ± 0.9	^c^ 3.0 ± 0.8	^a^ 2.8 ± 0.8
social support home	^a^ 3.1 ± 1.1	^a^ 3.3 ± 1.1	^b^ 3.5 + 1.0	2.6 + 0.7	^a^ 2.8 ± 0.9	^b^ 3.1 + 0.8
school support	^a^ 3.2 ± 1.1	^a^ 3.1 ± 1.0	^a^ 2.8 ± 1.1	2.6 ± 0.7	2.8 ± 0.9	^a^ 2.8 ± 0.9

Note: Physical Activity Questionnaire for Children (PAQ-C) [42]; measures of psychological variables; self-management strategies [37], perceived barriers to PA [47], outcome-expectancy value of PA [47], enjoyment of PA [37], self-efficacy [37], social support from both family and friends and the school [41]. There were no significant differences between any of the measures at baseline, after the intervention or at follow-up within each school (*p* > 0.05). There were between school differences ^a^ *p* < 0.05, ^b^ *p* < 0.01, ^c^ *p* < 0.001.
